# 肺腺癌患者在Atezolizumab治疗过程中出现假性进展的案例报道

**DOI:** 10.3779/j.issn.1009-3419.2019.06.10

**Published:** 2019-06-20

**Authors:** 雪 王, 艺 赵, 智伟 陈

**Affiliations:** 200030 上海，上海交通大学附属上海市胸科医院，肺部肿瘤中心 Shanghai Lung Cancer Center, Shanghai Chest Hospital, Shanghai Jiao Tong University, Shanghai, 200030, China

**Keywords:** 肺肿瘤, 免疫治疗, PD-L1, Atezolizumab, 假性进展, NSCLC, Immunotherapy, PD-L1, Atezolizumab, Pseudoprogression

## Abstract

肺癌是全球癌症相关死亡的最常见原因。根据病理类型的不同，肺癌可分为非小细胞肺癌（non-small cell lung cancer, NSCLC）和小细胞肺癌（small cell lung cancer, SCLC）。其中NSCLC约占所有肺癌患者的85%。免疫检查点抑制剂（immune checkpoint inhibitors, ICIPs）是一类针对程序性死亡受体-1（programmed cell death protein 1, PD-1）及其配体（programmed death-ligand 1, PD-L1）的抑制剂，已有研究结果显示ICIPs在许多不同的癌症中具有良好且持久的抗癌疗效，其中抗PD-L1单克隆抗体Atezolizumab（MPDL3280）正在实体瘤和恶性血液病中开展临床研究。据报道，假性进展是免疫治疗中可能出现的独特现象之一。本文中我们报道了1例晚期NSCLC接受免疫治疗后发生假性进展的病例，希望这一案例可以更好地帮助临床医生恰当评估免疫治疗的疗效，并作出最恰当的治疗决策。

## Abstract (en)

Lung cancer is the most common cause of cancer-related death worldwide. There are two classes of lung cancer: non-small cell lung cancer (NSCLC) and small cell lung cancer (SCLC). NSCLC represents approximately 85% of all lung cancer cases. Immune checkpoint inhibitors (ICIPs) are a class of inhibitors of programmed death-1 and programmed death-ligand 1. Preclinical studies have shown that ICIPs have shown good clinical efficacy and durable response in diverse cancers. Among them, atezolizumab (MPDL3280), an anti-PD-L1 monoclonal antibody, is being investigated as a potential therapy against solid tumors and hematologic malignancies in humans. Pseudoprogression is reported as one of the unique phenomena with immune therapeutic agents. Here we report case of a person with advanced NSCLC who developed pseudoprogression after receiving immunotherapy. We hope this case could help clinicians to make appropriate decision when assessing therapeutic effects of immunotherapy.

肺癌因其在全世界范围内的高发病率和高死亡率而备受关注^[1]^，近年来随着免疫治疗和靶向治疗的发展及“精准医疗”概念的提出，肺癌的治疗越来越追求个体化。现存肺癌相关治疗手段各有利弊，耐药性及毒副反应等问题不断给肺癌的治疗带来挑战。

免疫检查点抑制剂（immune checkpoint inhibitors, ICIPs）的问世使得肺癌的治疗产生重大的变革。程序性死亡受体-1（programmed cell death protein 1, PD-1）是一种表达于T细胞表面的免疫抑制分子，PD-1配体（programmed death-ligand 1, PD-L1）作为其配体广泛表达于多种肿瘤细胞表面，二者结合后可以启动T细胞的程序性死亡，使肿瘤细胞获得免疫逃逸。因此针对PD-1和PD-L1的靶向抑制剂能够抑制肿瘤细胞的免疫逃逸，增强抗肿瘤免疫，从而发挥抗肿瘤治疗作用^[2, 3]^。目前国内已开展多种针对该类单克隆抗体的临床研究。其中，PD-L1抑制剂Atezolizumab已进入Ⅲ期临床研究。

随着免疫治疗的开展，许多与之相关的问题相继出现。如何正确的评估免疫治疗疗效是肿瘤内科医生所面临的一大挑战。众所周知，免疫治疗后肿瘤可能出现短暂体积的增大或者病灶数量的增加，随后出现肿瘤的迅速缩小或者病情稳定（stable disease, SD），这种现象被称之为假性进展^[4]^。之前的研究报道显示，假性进展主要见于晚期黑色素细胞瘤患者，相比之下肺癌中少见^[5]^。针对假性进展病灶的组织活检结果往往提示炎性细胞的大量浸润及组织坏死。因此依据实体瘤治疗疗效评价标准（Response Evaluation Criteria in Solid Tumour, RECIST）1.1进行单纯影像学评估容易将假性进展错误判定为“疾病进展”。在一项关于71例接受抗PD-L1治疗并发生“疾病进展”的晚期NSCLC患者的研究中（依据RECIST标准），发现仅有5.6%的患者在被判定为“疾病进展”后会很快出现肿瘤的缩小，即出现了假性进展^[6]^。当假性进展与真性进展难以鉴别时，临床医生需要通过不断的经验积累以正确评估肿瘤的免疫治疗效果，以便更好地做出下一步抉择-继续免疫治疗，或者考虑其他方案。

本文中，我们将报道1例晚期非小细胞肺癌（non-small cell lung cancer, NSCLC）假性进展案例，该患者正在参加我们病区的一项在既往未接受过化疗的Ⅳ期非鳞状NSCLC患者中与卡铂或顺铂+培美曲塞相比评价Atezolizumab（MPDL3280A，抗PD-L1抗体）联合卡铂或顺铂+培美曲塞的开放性、随机化、Ⅲ期研究。

## 1 临床案例

2018年4月，一例56岁的男性患者无意间发现左腋窝肿块，如鸡蛋大小，轻微疼痛，活动度可，质地较硬。遂至当地医院完善胸部计算机断层扫描（computed tomography, CT）检查考虑：右肺上叶周围型肺癌伴纵隔及左腋窝淋巴结及双肾上腺转移可能性大。患者继续完善正电子发射计算机断层显像（positron emission tomography-computed tomography, PET-CT）及头颅增强核磁共振（magnetic resonance image, MRI）考虑：右肺周围型肺癌伴右肺门，纵隔，头颅、双侧肾上腺、左腋窝及右锁骨上多发淋巴结等部位多发转移。为进一步明确诊断，患者遂至我院肿瘤内科，完善检查，排除禁忌后全身麻醉下行超声内镜引导下的经支气管针吸活检术（endobronchial ultrasound guided tranbronchial needle aspiration, EBUS-TBNA），术后病理诊断为：肺腺癌，分期：C-T2N3M1c，Ⅳb期。酶标：CK（+）、TTF-1（+）、P40（-）、CD56（-）、NapsinA（+)。基因检测示：*EGFR*（-）、*ALK*（-）、*ROS1*（-）、*B-raf*（-）、*K-ras*（-）、*PD-L1*（肿瘤细胞80%+），并制定患者疗效评估依据：靶病灶（右上肺结节、双侧肾上腺占位、纵隔淋巴结及左腋下淋巴结）与非靶病灶（双肺、右侧胸膜、心包积液、前腹壁皮下结节、脑、右锁骨上淋巴结等）。2018年6月12日于我院放疗科行全脑放疗，并随即入组免疫课题于2018年6月28日起行一线培美曲塞、顺铂联合Atezolizumab方案治疗，每3周一个疗程，每个疗程的第1天用药。患者按照课题要求完成4周期前述方案治疗。在第一周期化疗结束10天后，患者自觉左腋窝淋巴结出现发热、红肿、疼痛等不适，随后左侧腋窝淋巴结逐渐缩小，并在两周期化疗结束时（7月底）基本消失。于2018年8月3日本院复查疗效，胸部CT及腹部CT提示：靶病灶（右肺上叶肿块、纵隔主动脉弓旁淋巴结）及非靶病灶（前腹壁下结节、心包积液）明显进展，病人自觉腹壁肿块红肿发热（突出于腹壁表面约5 cm），表面呈现紫红色。完善相关检查后，患者于2018年8月9日行超声引导下腹壁肿块穿刺术，术后病理回示：纤维组织中见少量异型小圆细胞，伴坏死，倾向恶性，酶标：CK（-）、TTF-1（少-）、P40（-）、CD56（+）、NapsinA（-），考虑疗效为疾病进展（progressive disease, PD）。但患者部分转移病灶（腋窝下淋巴结，双肾上腺占位，其余纵隔淋巴结）明显缩小，且自觉一般情况良好，综合考虑后，课题研究认为虽然该患者多个病灶的大小出现“增加”，但患者具有生存获益（一般情况好转），尚未达到需要终止治疗的程度，故继续该方案治疗。2018年8月20日起前腹壁下结节快速缩小，直至2018年9月7日第4周期化疗前，前腹壁肿块完全消失，表面皮肤凹陷（图 2）。2018年9月13日全身检查疗效评估为靶病灶PR（相比基线缩小37.2%），全身疗效评估为PR。

**2:**
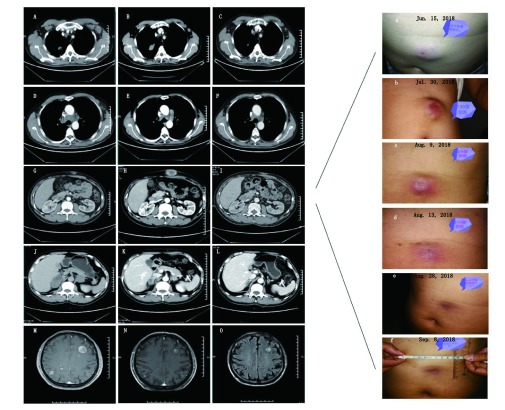
CT变化：基线水平（使用atezolizumab 2周前）；假性进展（治疗6周后）；肿瘤缩小（治疗12周后）。A-C：2018年8月3日胸部CT显示右肺上叶肿块（白色箭头）较基线水平明显增大。2018年9月13日，该肿块较前迅速缩小。治疗开始后左腋窝淋巴结持续缩小（红色箭头）；D-F：主动脉弓旁纵隔淋巴结（白色箭头）在治疗第6周时增大，第12周时明显减少。其余纵隔淋巴结体积在治疗后第6周显著减小（红色箭头）；G-I：前腹壁肿块（白色箭头）治疗第6周时出现，随后迅速增大，第12周时明显缩小；a-f：病人自行拍摄，前腹壁肿块肉眼外观变化，2018年7月30日该肿块最大；J-L：右肾上腺肿块第6周时明显缩小；第12周较第6周进一步缩小；M-O：第6周时脑转移肿块的数量及体积均减少，第12周较第6周变化不大 CT scans showing tumour response at baseline (2 weeks before initiation of atezolizumab), pseudoprogression (immediately after 6 weeks of treatment), and after tumour shrinkage (after 12 weeks of treatment). A-C: Chest CT images show the right superior lobe mass (white arrow) significantly increased in size on August 3, 2018 compared with the baseline. On September 13, 2018, the lesion shrunk significantly. Left axillary lymph nodes decreased in size since therapy (red arrow). D-F: The mediastinal lymph nodes (white arrow) near the aortic arch grew larger at week 6 and subsequently decreased definitely at week 12. Others significantly decreased their size at week 6 (red arrow). G-I: An anterior abdominal wall mass (white arrow) was detected at week 6, which was larger and subsequently smaller at week 12. a-f: The size change of the anterior abdominal wall mass, photographed by the patient himself. The anterior abdominal wall masssignificantly increased in size on July 30, 2018. J-L: Right adrenal mass shrunk significantly at week 6. M-O: The amount and volume of brain metastasis were both reduced at week 6 and week 8

## 2 讨论

我们报道了1例Atezolizumab治疗晚期肺腺癌期间发生的假性进展病例。在最初的治疗后，病人很快出现影像学上疾病进展，通过组织活检及随后的病情观察中我们最终明确了免疫疗法的疗效，判定其为假性进展。目前在中国，免疫疗法在抗NSCLC的治疗中取得很大进步，许多患者从中获益，但是一些非典型肿瘤反应的出现也给它的应用带来挑战，如延迟效应、假性进展、超进展和远隔效应^[7]^。

一般认为，假性进展并不是真正的肿瘤增殖，而是免疫治疗期间肿瘤内炎细胞浸润、水肿和坏死所致的肿瘤增大。此时影像学上可见肿瘤体积增大^[8, 9]^，伴或不伴临床生存的获益。其具体机制尚不明确。假性进展的表现形式多种多样，原发病灶^[10]^和转移病灶的增大（脑^[11, 12]^、肝^[13]^和肺^[14]^等部位）、新发病灶的出现（纵隔淋巴结^[14]^）及延迟效应^[15]^等都是常见的影像学特征。肺鳞癌和腺癌中均有假性进展案例的报道，但具体的发生比率尚不清楚。

**1:**
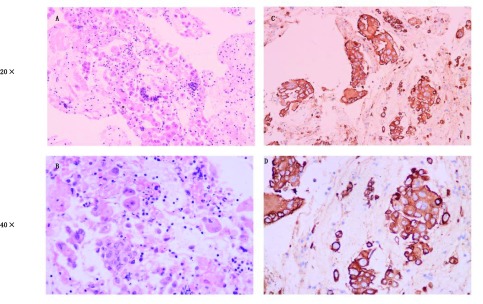
组织病理。A-B：患者初次诊断病检肺腺癌H & E染色（A：×20；B：×40）示：多形性肿瘤细胞浸润且有丝分裂数增加；C-D：免疫组化，CK阳性（C：×20；D：×40） Histological analysis. A-B: At initial diagnosis, H & E staining (A, ×20; B, ×40) shows pleomorphic tumour cell infiltration and increased mitotic figures in the biopsy sample. C-D: Immunohistochemistry is positive for CK (C: ×; D: ×40)

临床上，医生通过应用免疫治疗相关疗效评估标准及组织再活检等手段，不仅可以避免对患者疗效的错误评估，而且可以制定出更准确和更富有个体化的治疗方案。具体来说，实体瘤疗效评价指标-RECIST标准可能无法充分评估免疫治疗的疗效，目前国际上有几种新的评价标准，包括免疫相关反应标准（immune-related response criteria, irRC）和免疫相关的疗效评价指标（immune-related response evaluation criteria in solid tumors, irRECIST）等，实践证明，它们能够更准确地判断出接受免疫治疗的患者是否出现了疾病进展^[1, 7, 8]^；此外，针对“疾病进展病灶”的组织活检，若发现明显的免疫细胞浸润伴有局部组织坏死等病理特征，能充分证明假性进展的出现。

总之，在使用免疫药物期间，医生应密切监测患者全身病灶的变化，警惕假性进展的出现，这也许是药物发挥疗效的先兆。我们认为，必要时的组织活检及频繁的影像学监测能使接受免疫疗法的患者最大程度地获益。医务工作者通过对免疫治疗相关现象的报告和研究，能更好地指导患者进行抗癌治疗，同时也为科研工作者提供科学研究的方向。

**3:**
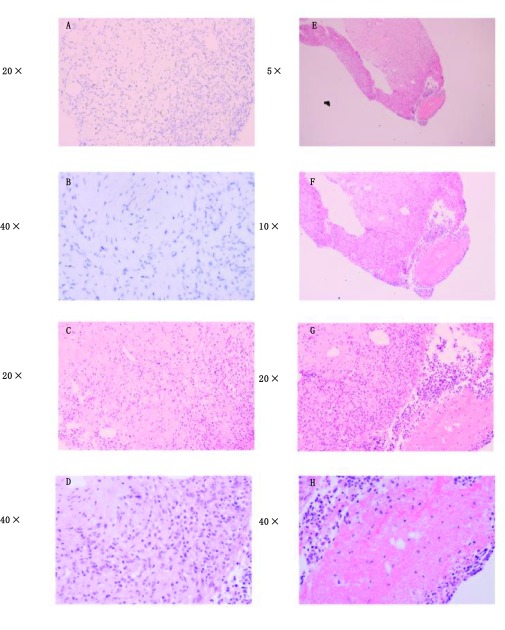
前腹壁肿块的组织活检。A-B：免疫组化，CK阴性（A：×20；B：×40）；C-H：H & E染色显示（C：×20；D：×40）：组织内少量浆细胞浸润以及明显的淋巴细胞浸润，伴有局部组织坏死（E-H） Tissue section of the anterior abdominal wall mass biopsy. A-B: Immunohistochemistry is negative for CK (A, ×20; B, ×40); C-H: H & E staining (C, ×20; D, ×40) shows few plasma cells and marked lymphohistiocytic infiltration with local tissue necrosis (E-H)
